# Digital twins techniques in the design of Alzheimer’s disease clinical trials: an application to the Internet‐Based Conversational Engagement Clinical Trial (I‐CONECT)

**DOI:** 10.1002/alz70859_100939

**Published:** 2025-12-25

**Authors:** Chao‐Yi Wu, Liu Chen, Steven E Arnold, Hiroko H Dodge

**Affiliations:** ^1^ Massachusetts General Hospital, Harvard Medical School, Boston, MA USA

## Abstract

**Background:**

With U.S. Food and Drug Administration (FDA)‐approved anti‐amyloid, partially disease‐modifying treatments now available, the ethical justification for randomly assigning patients to placebo has become controversial. The idea of twin‐controls (or virtual‐controls) has gained significant attention in recent years, with the aim of creating twin patient cohorts that can be used as a surrogate to evaluate the effects of treatment on a personalized level. While promising, the feasibility of digital twins techniques remains largely untested.

**Method:**

I‐CONECT is a multi‐site, single‐blind, randomized controlled trial (RCT) examining the effects of conversational interactions on cognition among socially isolated subjects aged ≥ 75 years (normal cognition; mild cognitive impairment). 186 participants were randomized into experimental or control groups. The experimental group engaged in video chats with study staff 4 times/week for 6 months, while the control groups received weekly 10‐minute phone calls. The current analysis focused on the efficacy‐shown Montreal Cognitive Assessment (MoCA; global cognition) and category fluency animals (CFA; language‐based executive function) at 6‐month follow‐up. Data from the National Alzheimer’s Coordinating Center‐Uniform Data Set (NACC‐UDS) were used to create digital twins for treatment participants through two methods. Method 1 involved twin mapping, matching participants with 1 to 20 twins who had similar demographic, biological, and social factors, and comparing change scores between each participant and their twins. Method 2 used direct modeling by building random forest models to predict change scores as if participants were assigned to a “usual care” control group. Effect sizes were compared between original and twin‐controls trials, as well as between the two methods.

**Result:**

Approximately 10% of NACC‐UDS participants (5,332 out of 50,259) were eligible for I‐CONECT. For parallel‐group designs, treatment effect sizes on MoCA closely aligned between original (β=1.67) and twin‐control (β=1.46‐1.97) trials when the Euclidean distance mapping was applied. Similar findings were found in CFA (original trial β=2.56; twin‐control trial β=2.31‐3.34). For single‐case, n‐of‐1 designs, methods 1 and 2 showed substantial agreement in identifying treatment responders (Cohen’s Kappa=1 for MoCA; 0.68 for CFA).

**Conclusion:**

Digital twins from publicly available datasets enhance the rigor of RCTs by providing mapped twins as controls for early‐phase dementia trials.

